# Stabilization of Oil-in-Water Pickering Emulsions by Surface-Functionalized Cellulose Hydrogel

**DOI:** 10.3390/gels10110685

**Published:** 2024-10-24

**Authors:** Inimfon A. Udoetok, Mohamed H. Mohamed, Lee D. Wilson

**Affiliations:** Department of Chemistry, University of Saskatchewan, 110 Science Place, Saskatoon, SK S7N 5C9, Canada; mohamed.mohamed@usask.ca

**Keywords:** Pickering emulsion, hydrogel, surface functionalized, cellulose, sonication, apolar oil, cellulose, biopolymer

## Abstract

An amphiphilic cellulose (**CLH**) hydrogel was synthesized via grafting of quaternary ammonium groups onto cellulose. The structural properties of **CLH** were characterized via Fourier transform infrared (FTIR)/^13^C solid-state NMR spectroscopy, elemental (CHN) analysis, particle size distribution (PSD), thermogravimetric analysis (TGA), and wettability was assessed through contact angle measurements. Pickering emulsions of apolar oils in water were prepared using variable weights of the **CLH** hydrogel as the stabilizing agent, along with different methods of agitation (mechanical shaking and sonication). The characterization results for **CLH** provide support for the successful grafting of quaternary ammonium groups onto cellulose to produce hydrogels. Different methods of agitation of an oil/water mixture revealed the formation of an *oil-in-water* (O/W) Pickering emulsion that was stable to coalescence for over 14 days. The resulting emulsions showed variable droplet sizes and stability according to the dosage of **CLH** in the emulsion and the agitation method, where the emulsion droplet size is related to the particle size of **CLH**. The addition of methyl orange (MO), a probe to evaluate the phase partitioning of the dye, had minor effects on the emulsion droplet size, and the emulsion prepared with 0.8 wt.% of **CLH** and agitated via sonication exhibited the smallest droplet size and greatest stability. This study is anticipated to catalyze further research and the development of low-cost and sustainable biopolymer hydrogels as stabilizers for tunable Pickering emulsion. Grafted cellulose materials of this type represent versatile stabilizing agents for foods, agrochemicals, and pharmaceutical products and technologies.

## 1. Introduction

Emulsions refer to heterogeneous systems where immiscible liquid phases coexist as the dispersed and continuous phases [1,2]. The dispersed phase usually exists in the form of droplets with diameters that often exceed 0.01 μm suspended in the continuous phase. Emulsions are formed either spontaneously, or through mechanical agitation, due to the high interfacial energy requirements of the dispersed droplets. This high interfacial area usually imparts thermodynamic and kinetic instability to emulsions, thus requiring the application of emulsifiers for stable formulations. Emulsifiers are amphiphilic species that are able to adsorb at the oil–water (O-W) interface and lower the interfacial tension between the phases [3]. The emulsifiers adsorbed at the O-W interface form a protective film between the droplets that resists their coalescence and phase separation, thus affording stable emulsions.

Emulsifiers are generally low-molar-mass surfactants or larger surface-active polymers [3,4]. However, due to toxicity concerns about the use of conventional surfactants in biomedical applications [2,5,6], the works of Ramsden [7] and Pickering [8] demonstrated the utility of solid particles of submicron or nanometre size as emulsion stabilizers due to their ability to adsorb strongly at the O-W interface [3,4,9]. These particle-stabilized emulsions became known as Pickering emulsions in recognition of the contributions of S. U. Pickering [10]. The ability of Pickering-based emulsifiers to support the retention of several of the basic properties of conventional emulsions lends support to their utility as alternative emulsifiers for many applications where the toxicity of surfactants poses a concern [2,5,11,12,13]. In addition to overcoming toxicity challenges, these emulsions have been the subject of considerable research due to their ability to address emulsion instability arising from gravitational separation (creaming/sedimentation), flocculation, coalescence, Ostwald ripening, and phase inversion [14,15]. Studies on Pickering emulsions have revealed the suitability of gold [16], biomaterials [17,18,19,20,21,22,23], latex [24], iron carbonyl [9], modified and pristine iron oxide [25,26,27,28,29], silica [24], titanium dioxide [30], and zinc oxide [31] as emulsion-stabilizing agents. The use of biomaterials as sustainable emulsifiers has generated continued research interest due to their low toxicity, low cost, availability, and renewable nature. Recent studies [11,17,32,33,34] have shown that biomaterials such as polysaccharides, proteins, fats, and oils are predominantly used as emulsifiers. However, these studies [11,17,32,33,34] indicate that cellulose in its modified form is a relatively common biomaterial for the stabilization of Pickering emulsions over other polysaccharides. This is attributed to the low cost, abundance, and non-toxicity of cellulose. As well, the ability of its modified forms to adsorb strongly at the O-W interface provides a mechanical barrier to droplet coalescence. Gong et al. [35] demonstrated that modified cellulose nanocrystals (m-O-CNCs) were better Pickering-emulsion-stabilizing agents than Tween-20, a traditional synthetic surfactant. They revealed that 1.5 g/L of m-O-CNCs was required to achieve a dispersed-phase volume fraction (DPVF) of 0.7, whereas 20 g/L of Tween-20 was used to achieve a similar effect. Other studies [19,20,21,22,23,33,34,36,37,38,39,40,41] also show that cellulose in various forms can be used as an emulsifier agent. Among the forms of cellulose employed as Pickering emulsion stabilizers, cellulose micro- and nanocrystals in modified and non-modified forms are popular [19,22,39,40,42,43,44,45]. By contrast, studies of “bulk” cellulose (micron-sized particles) and its modified forms have been scarcely reported [18,33,36,37,46,47]. While studies on the use of “bulk” cellulose and its modified forms are available in the literature, many reports are known for the stabilization of emulsions in apolar oils, such as hexane [18], kerosene [33], dodecane [36], paraffin oil [37], vegetable oil/kerosene [46], and soybean oil [47].

In this research, we demonstrate the efficiency of surface-functionalized cellulose (**CLH**) hydrogels as Pickering emulsifier agents for apolar oils with different hydrocarbon chain lengths (octanol and olive oil). The results of this study will contribute to the valorization of cellulose in several ways: (i) by facilitating a greater understanding of the effectiveness of modified cellulose as a Pickering emulsifier, (ii) by providing further insight into the general utility of cellulose materials as sustainable emulsifier agents, and (iii) by revealing the wider utility of cellulose materials for potential industrial applications, such as the production of antibacterial creams, personal care products, and drug delivery agents.

## 2. Results and Discussion

### 2.1. Characterization of Surface-Functionalized Cellulose (**CLH**)

#### 2.1.1. FTIR Studies

**Figure 1:**
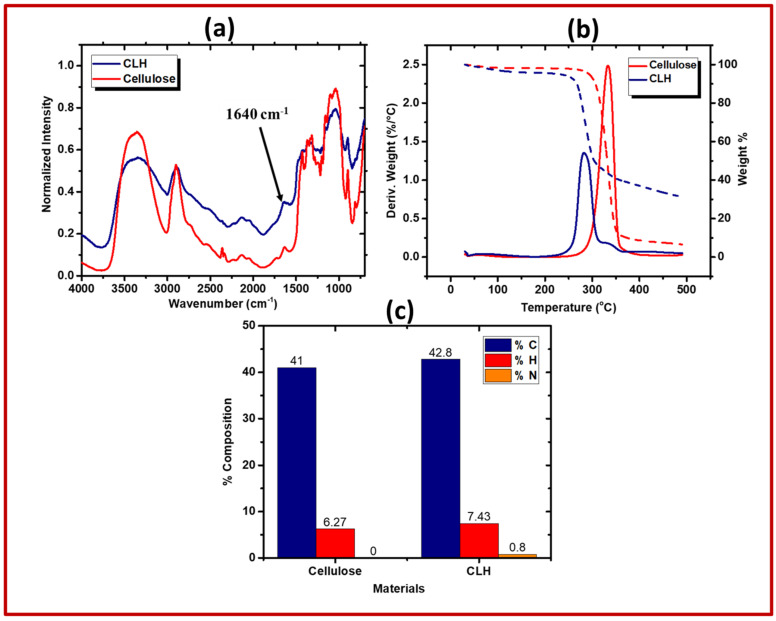
(**a**) FTIR spectra of cellulose and the **CLH** hydrogel, (**b**) DTG (solid line) and TG (dashed line) profiles of cellulose and the **CLH** hydrogel, and (**c**) CHN composition of cellulose and the **CLH** hydrogel.

FTIR spectroscopy is a technique that relies on variations in the absorption of IR spectral radiation by specific functional groups of a material to provide evidence of chemical modification [48,49]. The FTIR spectra of cellulose and **CLH** are displayed in Figure 1a. The spectra illustrate several features: a broad band at ~3000–3600 cm^−1^ due to intermolecular bonded OH and NH groups, C–H stretching at 2800–3000 cm^−1^, O–H/C–H bending at 1400–1300 cm^−1^, and C–O–H/C–O–C asymmetric stretching at 1000–1200 cm^−1^. The above spectral results show close agreement with reports on other IR studies of cross-linked and functionalized cellulose [18,50,51,52]. The spectra show that the C–H stretching band at 2800–3000 cm^−1^ becomes broadened, while the bands between 1300 and 1400 cm^−1^ are attenuated relative to cellulose. These effects may be ascribed to the modification of the –OH groups of cellulose via a grafting reaction, in agreement with another study [53]. A salient feature of the **CLH** spectrum is the spectral broadening and intensity of the band at 1640 cm^−1^, attributed to sorbed water. This feature provides support for the introduction of quaternary ammonium groups onto cellulose since such groups are hygroscopic and show favourable hydration properties [53,54,55].

#### 2.1.2. Thermogravimetric Analysis

Thermogravimetric (TG) profiles reveal the sample mass changes with temperature due to thermal decomposition, sublimation, vaporization, adsorption, desorption, and redox processes [49]. A plot of the TG profile (wt.%) versus temperature or the derivative TG (wt.%/°C) versus temperature (°C) in Figure 1b reveals a single thermal degradation event for cellulose, whereas two events occur for the **CLH** hydrogel, in agreement with another study [56]. The results for **CLH** show a lower temperature onset and a wider temperature range for thermal degradation versus cellulose. **CLH** shows lower mass loss relative to cellulose due to its greater thermal stability. The differences in the thermal degradation profiles of cellulose and **CLH** agree with the role of quaternization effects and concur with the broadened FTIR band at 1640 cm^−1^, which provides support for the grafting of quaternary ammonium groups onto cellulose. Previous studies [57,58,59] related to the thermal degradation of grafted polymers reveal that the degradation process begins with the surface-grafted domain of the polymers at lower temperatures, followed by the polymer backbone at higher temperatures. This trend concurs with the thermal degradation of 3-chloro-2 hydroxypropyl-trimethylammonium chloride (CHPTAC)-grafted cellulose, where the thermal degradation process commenced at lower temperature relative to pristine cellulose [56]. The change in the thermal degradation profile of **CLH** may be a result of the alteration of the hydrogen bond network and thermal stability due to the presence of grafted quaternary ammonium groups on the cellulose surface.

#### 2.1.3. Elemental (CHN) Analysis

Elemental analysis provides information on the composition (wt.%) of elements such as C, H, and N, along with heteroatoms like sulphur and halogens. The CHN composition of cellulose and **CLH** hydrogel is presented as a bar graph in Figure 1c. The results show that the composition (wt.%) of carbon and hydrogen in **CLH** is higher relative to cellulose. **CLH** contains N atoms, while cellulose has zero (wt.%) nitrogen, in agreement with its biopolymer structure. The differences in the CHN content of cellulose and **CLH** relate to the grafting of quaternary ammonium groups onto cellulose, which concurs with the results from the IR spectra and TGA profiles [60,61].

**Scheme 1:**
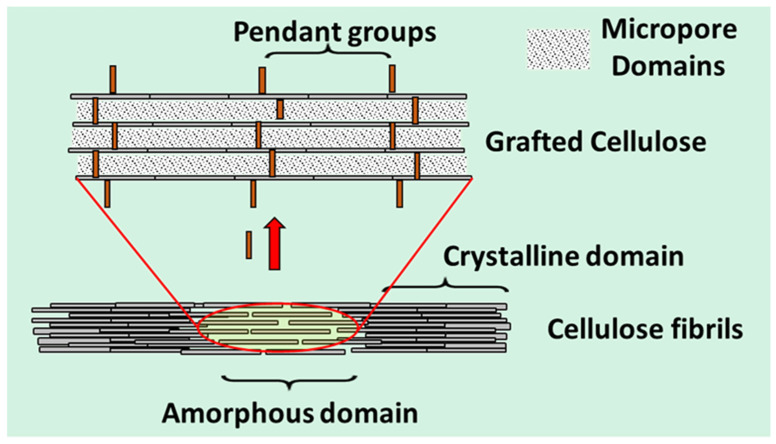
Schematic illustration of the grafting of cellulose by GTMAC.

Based on Equation (1), the degree of substitution (DS) of quaternary ammonium groups on cellulose was calculated as 0.1, according to the N content of **CLH** from the CHN analysis. This implies that about 10% of the approximately 30% accessible hydroxyl groups of cellulose were substituted by quaternary ammonium groups. Quaternization likely introduces defects that attenuate H-bonding among the fibril bundles of cellulose, resulting in a more amorphous structure (cf. Scheme 1). Song et al. [54] reported values of DS between 0.2 and 0.36 for cellulose upon quaternization with CHPTAC in NaOH/urea aqueous solutions. The CHPTAC materials were soluble in water, whereas the **CLH** material prepared herein is insoluble in water. These observations and the DS values provide support that partial pillaring of cellulose occurs upon quaternization, as depicted by the general illustration in Scheme 1.

#### 2.1.4. ^13^C CP-MAS Solid-State NMR Studies

**Figure 2:**
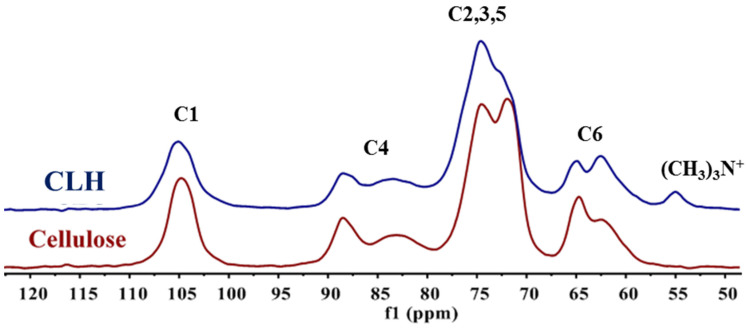
^13^C CP-MAS solid NMR spectra of cellulose and **CLH**.

Solids ^13^C NMR spectra obtained with CP-MAS afford high-resolution spectra that enable the structural characterization of modified biopolymers. Such information includes the attenuation or enhancement of the ^13^C signatures of the groups involved in the modification process [49]. Figure 2 presents the ^13^C CP-MAS NMR spectra of cellulose and the **CLH** hydrogel. The results show that the ^13^C signatures of pristine cellulose and **CLH** reside between 55 and 105 ppm, which provides support for the grafting reaction and is in agreement with the preservation of the cellulose carbon skeletal structure [62,63,64]. The successful grafting of quaternary ammonium groups onto cellulose is further affirmed by the greater line broadening of existing spectral lines, along with new signatures at 55.0 ppm attributed to (CH_3_)_3_N^+^ and ca. 77 ppm, which relate to the CH_2_ groups from the grafting agent (GTMAC) [62,64]. The significant line broadening of the ^13^C signatures of the quaternary ammonium groups aligns with the increased amorphous character of cellulose in **CLH** (DS = 0.1). The line broadening and appearance of new resonance lines in the spectra of **CLH** concur with the electrostatic effects of surface modification, along with the variable thermal degradation profiles, CHN results, and FTIR results in Section 2.1.1, Section 2.1.2, Section 2.1.3 and Section 2.1.4.

#### 2.1.5. Particle Size Distribution (PSD)

**Figure 3:**
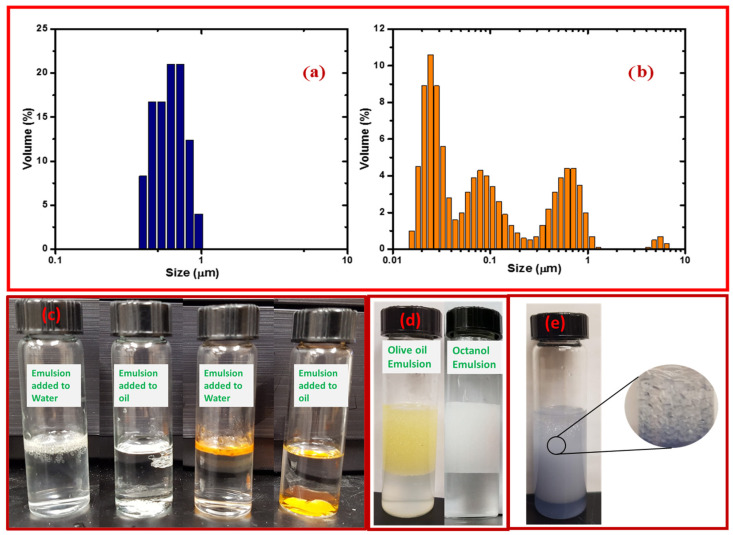
Particle size distributions of **CLH** after (**a**) 10 min of sonication and (**b**) 24 h of sonication; (**c**) the dilution test for octanol and olive oil emulsions; (**d**) pictures of the stable olive oil and octanol emulsions one year after preparation; (**e**) a picture of the octanol emulsion with a water-soluble dye (reactive black 5).

Figure 3a,b shows the particle size distribution of **CLH** hydrogel after different sonication intervals. The results reveal that the particles of **CLH** were unimodal without sonication, whereas the distribution became polymodal after 10 min and 24 h of sonication. The mean particle size of **CLH** is given for the various treatments: ~17 µm (without sonication), 14 nm (10 min of sonication), and 1 nm (24 h of sonication). The above results agree with the values obtained independently for modified cellulose [65] and starch particles [66].

### 2.2. Characterization of Emulsions

#### 2.2.1. Determination of Emulsion Type

**Scheme 2:**
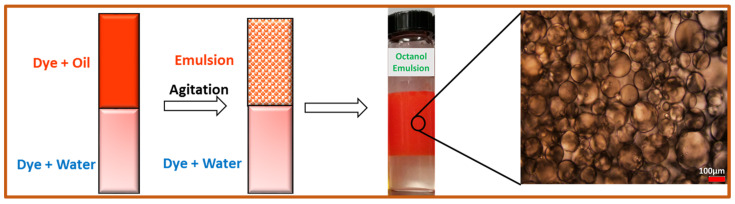
An illustration of dye solubility in the continuous and dispersed phases of the O/W emulsion.

The emulsion type was determined through cobalt chloride and dilution tests. The results reveal that the octanol and olive oil emulsions were the *oil-in-water* (O/W) type. In the cobalt chloride test, strips of filter paper soaked in pink cobalt chloride turned blue after immersion in the emulsions. The test shows that water is the continuous phase while oil droplets serve as the dispersed phase. The dilution test results (cf. Figure 3c) affirm oil as the dispersed phase, as diluting a small quantity of the emulsion in water resulted in the greater dispersion of the emulsion droplets, in agreement with similar studies [67,68]. Similarly, the yellow colour of the emulsion (cf. Figure 3d) prepared using yellow olive oil also affirms water as the dispersed phase for the emulsions. These results agree with the light microscopy images of the emulsion (cf. Figure 6), where the colour of methyl orange (MO) was more pronounced in the emulsion droplets (cf. Scheme 2) than in the water phase.

Previous reports [69,70] on the characterization of emulsions via dye solubility reveal that the dye colouration of the dispersed phase determines the colour of an emulsion. This agrees with the results presented in Section 2.2.1 of this study (cf. Figure 3e). FTIR and ^13^C NMR spectral characterization reveals that **CLH** may exhibit greater hydrophilicity due to the presence of quaternary ammonium groups on its surface. Particles with greater amphiphilic character and dispersibility tend to stabilize O/W emulsions, in agreement with the results of this study and other reports [47,71]. This occurs because particles with greater hydrophilicity are predominantly wetted by the aqueous phase, whereas oil resides on the concave side of the interface, and water is localized on the convex side. In the case of **CLH**, which does exhibit irregular shapes and sizes, the formation of an O-W emulsion may be due to a combination of factors, such as hydrophilicity and molecular structure, that enhance its adsorption at the O-W interface, along with the formation of a three-dimensional network in the continuous phase [72]. Utsel et al. [73] reported θ-values of 38°, 46°, and 65° for various modified cellulose materials, which support the formation of an O/W emulsion by functionalized cellulose, as described in Section 2.2.1.

#### 2.2.2. Wettability of Surface-Functionalized Cellulose

**Figure 4:**
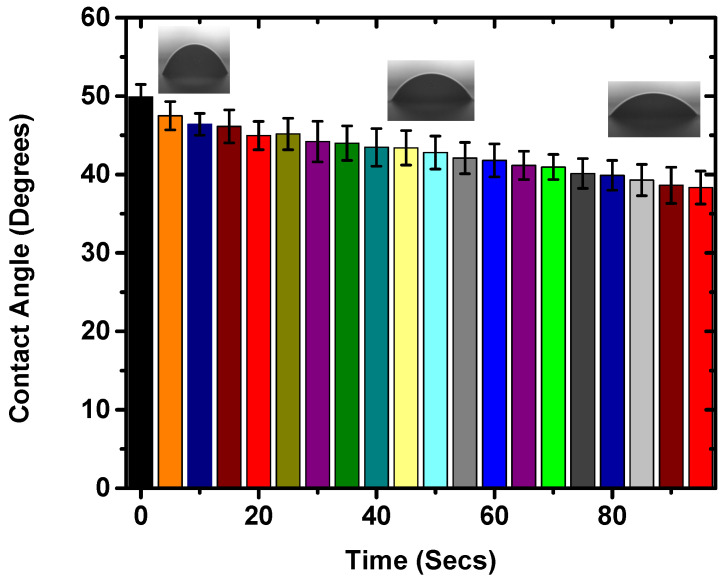
The dynamic contact angle of the **CLH** hydrogel.

The wettability of **CLH** determines its position at the oil–water interface, which was evaluated by measuring the contact angle (θ) values, as presented in Figure 4. The θ-values reveal that the initial contact angle of **CLH** is below 90° (θ = 49.9 ± 1.6°), in agreement with a related study [73]. Previous reports [74,75] of contact angle measurements reveal that the θ-value of a surface is affected by surface roughness, heterogeneity, and particle size and shape. The results indicate a linear decreasing trend for the θ-values of **CLH** with increasing time, where the recorded θ-value was 38.3 ± 2.1° after 95 s.

**Scheme 3:**
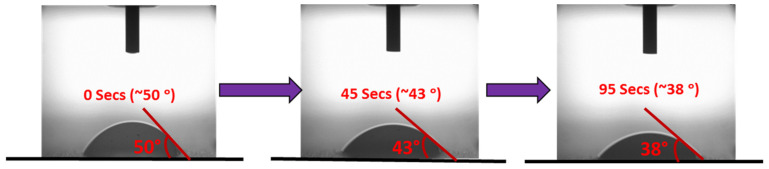
Images showing a decrease in water contact angle for the **CLH** system.

The above results reveal that the quaternization of cellulose confers greater surface roughness, heterogeneity, and porosity to the **CLH** hydrogel relative to pristine cellulose. The amphiphilic properties of **CLH** contribute greater hydrophilicity and interfacial water compatibility of the hydrogel. In turn, the imbibition of water by the pores of the film occur, as affirmed by a decrease in the water θ-values with increasing time (cf. Scheme 3 and Figure 4). The observed trend supports the effectiveness of **CLH** in stabilizing *oil-in-water* emulsions. Particles with greater hydrophilicity undergo favourable wetting by the aqueous phase, resulting in a lower θ-value for water [76].

#### 2.2.3. Size of Emulsion Droplets

**Figure 5:**
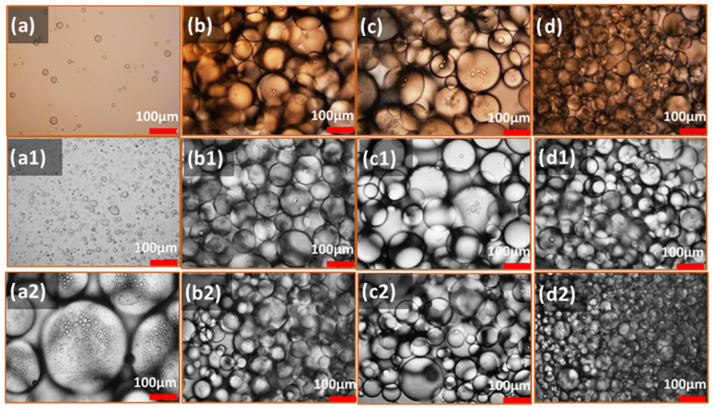
Optical microscopy images of emulsions prepared with (**a**) 2 mg of **CLH** + dye and shaking, (**a1**) 2 mg of **CLH** and shaking, (**a2**) 2 mg of **CLH** and sonication, (**b**) 4 mg of **CLH** + dye and shaking, (**b1**) 4 mg of **CLH** and shaking, (**b2**) 4 mg of **CLH** and sonication, (**c**) 6 mg of **CLH** + dye and shaking, (**c1**) 6 mg of **CLH** and shaking, (**c2**) 6 mg of **CLH** and sonication, (**d**) 8 mg of **CLH** + dye and shaking, (**d1**) 8 mg of **CLH** and shaking, (**d2**) 8 mg of **CLH** and sonication.

The size of emulsion droplets was determined via optical microscopy (cf. Figure 5) and measurements of the droplet size with ImageJ 1.52 software. The results reveal that the mass of **CLH,** as well as the nature of agitation, affects the emulsion droplet size. By contrast, the addition of MO to the emulsion had minor effects on the droplet size (cf. Figure 5a–d). The results also show that the emulsions are polydisperse in nature, where the droplet size ranges between 148 and 269 µm for emulsions prepared via shaking. This trend concurs with droplet sizes above 100 µm reported by Chevalier and Bolzinger [6]. The O/W Pickering emulsions in [6] were prepared via gentle mechanical mixing, hand shaking, or a rotating blade stirrer. The large emulsion droplet sizes witnessed in this study may be related to the large particle size of **CLH** and the aggregation of **CLH,** along with the effects from the type of agitation method. This trend agrees with a related study [66] where starch particles (<10 µm) were better emulsion-stabilizing agents relative to larger starch particles (>10 µm). The authors posited that larger starch particles (>10 µm) did not stabilize the emulsions because of aggregate formation and sedimentation due to gravity. Overall, the results show that the emulsion prepared with 8 mg of **CLH** via sonication exhibits the smallest droplet size, consistent with the expected acoustic cavitation effects, which yields a reduction in the particle size of **CLH** (cf. Figure 3b), as well as more efficient mixing and surface coverage effects. On the other hand, high surface coverage supports greater adsorption at the O-W interface due to the very high interfacial energy of adsorption exhibited by Pickering particles at an interface, which confers greater stability to the Pickering emulsions. The cavitation effect, supported by a reduction in the particle size of **CLH** from 17 µm to a range of 0.3–1 µm after 10 min of sonication, enhances the emulsion stability. Similar studies [6,77] on the effects of particle concentration on the stability of Pickering emulsions revealed that particles can either stabilize or destabilize emulsions according to the particle adsorption vs. droplet formation rate. At low surface coverage, a destabilizing effect occurs due to incomplete surface coverage, leading to droplet–droplet bridging, in agreement with the results presented herein. It is noted that a 2 mg dosage of **CLH** is too low for the formation of a stable emulsion (cf. Figure 5a–c).

#### 2.2.4. Stability of Emulsions

The stability of emulsions by dispersed particles largely depends on their wettability by the two liquids used for emulsion preparation, including the ability of the particles to adsorb irreversibly at the oil–water interface [76,78]. Emulsion stabilization requires 180° < θ > 0° and very high desorption energy, where the position of particles at the *oil–water* interface is described by the contact angle (θ), which relates to the desorption energy according to Equation (1):

(1) $$\Delta G_{d}=\pi r^{2}\gamma_{ow}(1-|Cos\;{\theta |}^{2})$$

where ∆*G_d_* represents the desorption energy of the particles, γ*_ow_* is the *oil–water* interfacial tension, *r* is the radius of the particle, and *θ* is the contact angle.

A θ-value of 90° indicates that a particle is equally immersed in both liquids, while θ < 90° indicates a more hydrophilic surface, which undergoes wetting by the aqueous phase. A θ-value above 90° relates to a particle with an apolar character that is wetted mainly by the oil phase. For hydrophilic particles, the large contact area (A) with water supports the stabilization of an O/W emulsion. However, lipophilic particles show a curvature toward the aqueous phase that results in favourable *water-in-oil* (W/O) emulsion formation [76].

**Figure 6:**
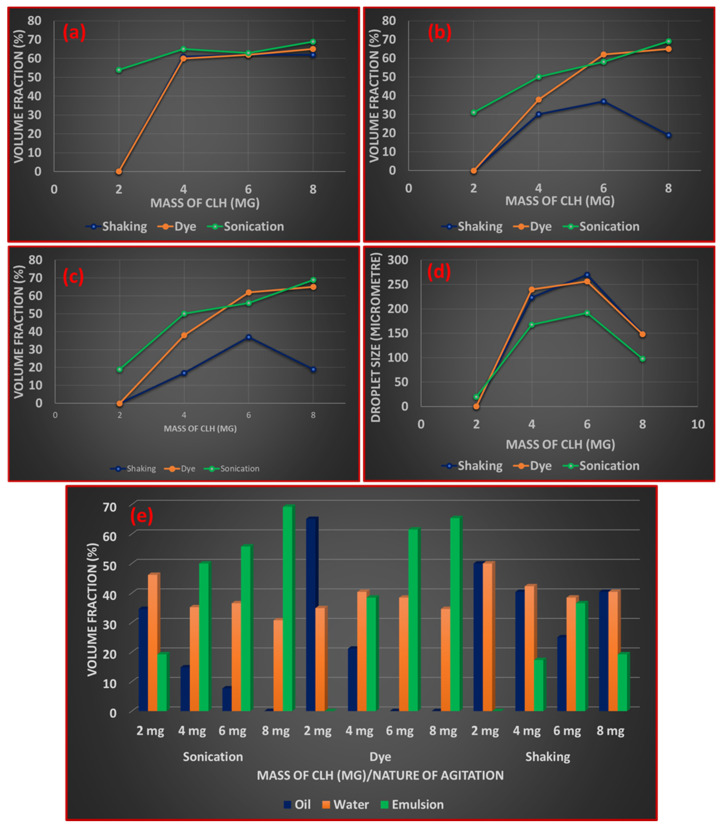
Stability of O/W emulsions: (**a**) 0 days after preparation, (**b**) after 7 days, (**c**) after 14 days. (**d**) The effect of particle concentration on emulsion droplet size and (**e**) volumes of water, oil, and emulsion 14 days after emulsion preparation.

The kinetic stability of the emulsions was assessed by measuring the initial volumes of water, oil, and emulsions, followed by measurements every 7 days over a 14-day period (cf. Figure 6a–c,e). The results show that all emulsions were initially stable to creaming and coalescence, except those prepared with 2 mg of **CLH**, along with shaking and dye addition (cf. Figure 6a). The lowered stability relates to the coalescence of the emulsion droplets due to the low dosage and large particle size of **CLH**, along with the shear applied during emulsion preparation. The low dosage, the large particle size of **CLH**, and the nature of shear stress cause inadequate adsorption of the particles at the *oil–water* interface, which leads to a slight lowering of the interfacial energy at the O/W interface. The role of shear effects is evidenced by the initial stability of the emulsion prepared with 2 mg of **CLH** and agitated via sonication. Acoustic cavitation effects due to sonication may enhance the formation of smaller emulsion droplets, as noted by the emulsion droplet-size results. After 7 days (cf. Figure 6b), the stability tests reveal that the emulsions prepared with 8 mg of **CLH** and agitated via sonication and 8 mg of **CLH** with dye and agitated via shaking are similar. By contrast, the emulsion prepared with 2 mg of **CLH** material and sonication was the least stable. After 14 days (cf. Figure 6c,e and Figure 3b), the stability of other emulsions decreased, while the stability of emulsions prepared with 8 mg of **CLH** and agitated via sonication and 8 mg of **CLH** with dye and agitated via shaking was unchanged. Figure 6e shows that the volume of emulsions increased as the mass of **CLH** increased, whereas the volumes of water were lower for a higher mass of **CLH**, except for the emulsion prepared by shaking. The result in Figure 6e reveals that some of the emulsions had become unstable due to creaming caused by their thermodynamic instability. The above is related to the effects of gravity on the particles due to their large sizes, in agreement with the disparity in the emulsion droplet size, which did not decrease with an increase in the mass of **CLH** as expected. The stability of the emulsions with an 8 mg **CLH** dosage compares favourably with other polysaccharide-based emulsion stabilizer particles (cf. Table 1). Generally, the emulsion stability increases with decreasing particle size and increasing particle dosage, as demonstrated herein, along with other reports [76,79,80]. The above results show that while 8 mg of **CLH** may have provided adequate surface coverage and efficient adsorption at the *oil–water* interface, the addition of MO dye may have enhanced the adsorption of the particles at the interface. MO (pK_a_ 3.46) exists as a cationic dye in acidic conditions (pH < pK_a_), which enhance dye binding to cellulose. Hence, a greater adsorption of the resulting dye–cellulose complex occurs at the *oil–water* interface. Panic et al. reported the optimal adsorption of MO by cellulose at pH 6.0, in agreement with the greater stabilization of the emulsions by the cellulose–MO composite. The results also show that acoustic cavitation enhanced the reduction of the emulsion droplet sizes, as well as the emulsion stability.

## 3. Conclusions

A **CLH** hydrogel was prepared through the successful grafting of quaternary ammonium groups onto cellulose, as evidenced by TGA, CHN, and spectral (FTIR/^13^C solids NMR) characterization results. The **CLH** hydrogel, used as a Pickering emulsifier with different agitation methods (e.g. mechanical shaking and sonication), produced *oil-in-water* (O/W) emulsions with variable droplet sizes. The O/W emulsions were temporally stable for 14 days, as demonstrated by complementary results reported herein. The emulsion droplet size was largely affected by the particle size (17 µm) and concentration of **CLH** in the emulsion, along with the nature of the shear force applied during emulsion preparation. **CLH** (8 mg) in the emulsions and agitation via sonication resulted in systems with the smallest droplet size and optimal stability. This trend concurs with a greater shear effect of sonication, which impacts the particle size distribution of **CLH**. The addition of MO, a dye with partial solubility in water and greater solubility in the oil phase, revealed the enhanced stability of the emulsions, with minor effects on the emulsion droplet size. The surface-functionalized cellulose (**CLH**) hydrogel reported herein is a sustainable alternative to synthetic surfactants, such as Tween-20 and other polyoxyethylene-based emulsifiers. It also offers a substitute for nanomaterials like nanocrystalline cellulose. This study is one of the few reports that describe the modification of “bulk” cellulose for its use as a Pickering emulsion stabilizer. We anticipate that a greater understanding of the structure–function relationships of surface-functionalized cellulose and its interfacial properties at the *oil–water* interface will draw attention to further research on the use of “bulk” cellulose. The use of such modified cellulose will overcome the need to utilize nanoparticle cellulose with solid particles at the micron scale and above. The findings of this study will further contribute to the valorization of nature’s most abundant biopolymer through its potential utility in diverse types of colloid formulations. Along these lines, further research is anticipated toward the development of low-cost and versatile biopolymer materials as tunable Pickering emulsifiers for stabilizing colloidal systems.

## 4. Materials and Methods

### 4.1. Materials

Cellulose (medium fibre from cotton linters), sodium hydroxide, glycidyl trimethyl ammonium chloride (GTMAC), olive oil, hydrochloric acid (HCl), methyl orange (MO), reactive black 5, ACS-grade acetone, and octanol were obtained from Sigma Aldrich (Saint Louis, MO, USA). All materials were used as received without further purification.

### 4.2. Synthesis of Surface-Functionalized Cellulose

Synthesis of surface-functionalized cellulose (**CLH**) was carried out using 2 g of “bulk” cellulose in 15 mL of 10% NaOH at 65 °C for 3 h, followed by the dropwise addition of 10 mL of glycidyl trimethyl ammonium chloride (GTMAC). The reaction mixture was stirred for at least 6 h before the separation of the final product via vacuum filtration. The resulting functionalized cellulose was washed with cold Millipore water, followed by ACS-grade acetone, and dried at 60 °C. Excess and unreacted reagents were removed via Soxhlet extraction for 24 h with HPLC-grade acetone, followed by drying in a vacuum oven at 60 °C for 12 h. The resulting **CLH** material was ground in a mortar and pestle and sieved with a 40-mesh sieve.

### 4.3. Characterization Studies

#### 4.3.1. Fourier Transform Infrared (FTIR) Spectroscopy

The FTIR spectra of **CLH** and pristine cellulose were obtained using a Bio-RAD FTS-40 IR spectrophotometer (Bio-Rad Laboratories, Inc., Philadelphia, PA, USA). The sample and pure spectroscopic-grade KBr were mixed in a weight ratio of 1:10 via co-grinding in a small mortar with a pestle before analysis. DRIFT (Diffuse Reflectance Infrared Fourier Transform) spectra were obtained in reflectance mode at 293 K with a resolution of 4 cm^−1^ over a 400–4000 cm^−1^ spectral range. Several scans were recorded and corrected against a background of pure KBr.

#### 4.3.2. Thermal Gravimetry Analysis (TGA)

The thermal stability of cellulose and **CLH** was determined using a TA Instruments Q50 TGA system (New Castle, DE, USA). The instrument was operated under the following conditions: a heating rate of 5 °C min^−1^ up to 500 °C and nitrogen as the carrier gas. The results are displayed as first-derivative (DTG) plots of weight with temperature (wt.%/°C) against temperature (°C).

#### 4.3.3. Carbon, Hydrogen, and Nitrogen (CHN) Analyses

The elemental compositions (carbon, hydrogen, and nitrogen) of **CLH** and cellulose were obtained using a Perkin Elmer 2400 CHN Elemental Analyzer (PerkinElmer, Inc., Waltham, MA, USA) with a combustion oven above 925 °C and a reduction oven operating above 640 °C. Purging of the instrument was achieved using a gas mixture of pure oxygen and helium, while acetanilide was used as the calibration standard. The results were obtained in triplicate with an estimated precision of ±0.3%. The degree of substitution (DS) of quaternary ammonium groups on cellulose was determined from the nitrogen content [98] according to Equation (2):

(2) $$DS=\frac{162\frac{g}{mol}\times N\%}{14\frac{g}{mol}\times 100-151.63\frac{g}{mol}\times N\%}$$

N% is the nitrogen wt. content of **CLH**; 162 g/mol is the formula mass of the anhydroglucose unit; 14 g/mol is the molar mass of N; and 151.63 g/mol is the molar mass of GTMAC.

#### 4.3.4. Solid-State ^13^C NMR Spectroscopy

The ^13^C solid NMR spectra of **CLH** and cellulose were acquired by employing a Bruker AVANCE III HD spectrometer (Bruker Bio Spin Corp., Billerica, MA, USA) furnished with a 4 mm DOTY CP-MAS (cross-polarization with magic angle spinning) solid NMR probe operating at 125.8 MHz (^1^H frequency at 500.2 MHz). The spectra were obtained with a spinning speed of 10 kHz, a ^1^H 90° pulse of 3.5 µs, a contact time of 0.75 ms, and a ramp pulse on the ^1^H channel. NMR acquisition parameters include a MAS rate of 10 kHz, a ^13^C 90° pulse of 3.15 µs, and a 25 kHz SPINAL-64 ^1^H decoupling during acquisition. A total of 5860 scans were accumulated for the samples, with a recycle delay of 2 s. During acquisition, all experimental data were recorded using a 71 kHz SPINAL-64 decoupling sequence, while the chemical shifts were referenced to adamantane at 38.48 ppm (low field signal).

#### 4.3.5. Contact Angle Measurements

The water contact angle (θ) of **CLH** was measured with a modified stereo microscope with a horizontal view path coupled with a Nikon Cool Pix 8400 camera (Nikon, Brisbane, Australia). The high-resolution images were analyzed using the Low Bond Axisymmetric Drop Shape Analysis Model (LB_ADSA) plugin within the ImageJ 1.52 software. A film was prepared by shaking 50 mg of **CLH** in 20 mL of Millipore water until a suspension was formed. The suspension was poured onto a glass slide (1 cm × 5 cm), followed by air drying. Images of 10 μL of water droplets were taken from the slide at 5 s intervals. This was repeated three times, and the contact angle (θ) was recorded as an average of the three replicate measurements.

#### 4.3.6. Particle Size Distribution Measurement

The particle size distribution of **CLH** was determined with a Malvern Zetasizer Nano ZS instrument (Malvern Instruments Ltd., Malvern, Worcestershire, UK). The sample preparation involved the dispersion of ~10 mg of CLH in 2 mL of millipore water, followed by sonication for 10 min or 24 h. Then, 75 µL of the dispersion was injected into the sample holder, and the measurements were obtained in triplicate at constant pH.

### 4.4. Preparation of Emulsions

**Scheme 4:**
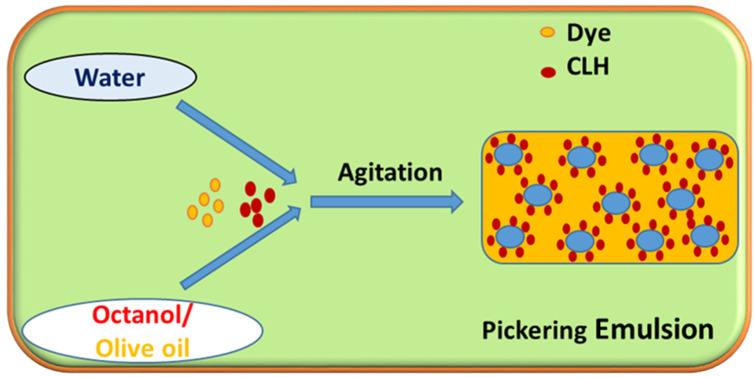
Schematic illustration of the *water-in-oil* (W/O) emulsion preparation method.

The emulsions were prepared according to the illustration in Scheme 4, where the details are as follows: variable weights of **CLH** (2, 4, 6, and 8 mg) were added to 30 mL vials. To the same vials, 10 mL of octanol was added, followed by 10 mL of water, with approximately 3 mg of methyl orange (MO) dye for some of the emulsions. The oil/water/**CLH** mixture was agitated using either ultrasonication or a horizontal mechanical shaker table. Another emulsion was prepared using 8 mg of the functionalized cellulose material, 10 mL of olive oil, and 10 mL of Millipore water and agitated via sonication. This was prepared to evaluate the effect of the oil chain length on the emulsion stabilization properties of **CLH**.

#### 4.4.1. Characterization of Emulsions

##### Determination of Emulsion Type

The emulsion type was determined using the dilution test, where an emulsion sample was added to water and oil separately. The miscibility of the emulsion in either oil or water was used to determine whether the emulsion was an *oil-in-water* (O/W) or a *water-in-oil* (W/O) emulsion.

#### 4.4.2. Determination of Emulsion Droplet Sizes

An IX83 Olympus light microscope (Olympus, Hamburg, Germany) was used to observe the emulsion droplets; emulsions were agitated gently in a vial before analysis to ensure homogeneity. A small drop of the sample was placed on a microscope slide, and the microstructure of the emulsion was observed with an optical microscope. Variable magnifications of 40× and 10× were used, and 5 images were obtained for each sample. The average droplet size was obtained by measuring 20 emulsion droplets each from 3 images using ImageJ 1.52 software.

## Figures and Tables

**Table 1:** Summary of some polysaccharides used as Pickering-emulsion-stabilizing agents.

S/N	Particle	Dosage	Oil Phase	Emulsion	References
1	**CLH**	0.2–0.8 wt% ^†^	Olive oil	O/W	This work
2	**CLH**	0.2–0.8 wt% ^†^	Octanol	O/W	This work
3	SNP	1 wt% ^†^	Undecanol	O/W	Tan et al., 2012 [81]
4	LNPs	0.2 wt% ^†^	Olive oil	O/W	Shimoni et al., 2013 [82]
5	Chitosan	0.1 wt% ^†^	Paraffin, hexane, toluene, dichloromethane	O/W	Liu et al., 2012 [83]
6	MFC	0.7 wt% ^†^	Soybean oil	O/W	Winuprasith and Suphantharika, 2013 [84]
7	SNCs	0.02–6 wt% ^†^	Paraffin	O/W	Li et al., 2012 [85]
8	CNCs	0.5 wt% ^†^	Rice bran oil	O/W	Angkuratipakorn et al., 2017 [86]
9	Starch particles	1–3 wt% ^†^	Tetradecane	O/W	Yusoff and Murray, 2011 [87]
10	Grafted CNCs	0.05–0.5 wt% ^†^	Heptane	O/W	Zoppe et al., 2012 [88]
11	Starch particles	0.5–5 wt% ^†^	Soybean oil	O/W	Song et al., 2015 [89]
12	Amorphous cellulose	0.07–1.10 wt% ^†^	Dodecane	O/W	Jia et al., 2015 [72]
13	CNPs	1% *w*/*w* *	Corn oil	O/W	Tzoumaki et al., 2011 [90]
14	MCC	1.5% *w*/*w* *	Sunflower oil	O/W	Kargar et al., 2012 [91]
15	Modified starch	1.5% *w*/*w* *	Sunflower oil	O/W	Kargar et al., 2012 [91]
16	CNCs	0.1–1.5%	Dodecane	O/W	Gestranius et al., 2017 [92]
17	CNCs	0.1%	Hexadecane	O/W	Capron and Cathala, 2013 [93]
18	CCMC, EC, and HPMC	2–3% *w*/*w*	-	O/W	Duffus et al., 2016 [94]
19	Acetylated CNCs	0.5–1.0 wt%	Dodecane, olive oil and paraffin	O/W	Torlopov et al., 2021 [95]
20	BC, CNCs, and CNFs	0.05–5.0 wt%	Olive oil	O/W	Lu et al., 2020 [96]
21	SPRC, OSA-SPRC, and Tween 80	0.25–1.75% *w*/*v*	Medium chain triglyceride	O/W	Xie et al., 2020 [97]
22	TOCN, TOChN, and DEChN	0.05–0.6 wt%	Soybean oil	O/W	Liu et al., 2023 [21]

* Calculated based on the weight of the particles relative to total oil/aqueous weight. ^†^ Calculated based on the weight of particles in the aqueous dispersion. CNCs—cellulose nanocrystals; SNCs—starch nanocrystals; MFC—microfibrillated cellulose; MCC—microcrystalline cellulose; CNPs—chitin nanoparticles; LNPs—lactoferrin nanoparticles; SNP—starch nanosphere; **CLH**—quaternized cellulose; CMCC—colloidal microcrystalline cellulose; EC—ethylcellulose; HPMC—hydroxypropyl methyl cellulose; BC—bacterial cellulose; CNF—cellulose nanofibres; SPRC—sweet potato residue cellulose; OSA—octenyl succinic anhydride.

## Data Availability

The original contributions presented in the study are included in the article.
